# Supplementary material for: A UMLS-based spell checker for natural language processing in vaccine safety
Source: BMC Med Inform Decis Mak. 2007 Feb 12;7:3. doi: 10.1186/1472-6947-7-3 (PMC1805499; doi:10.1186/1472-6947-7-3)
Supplement: Additional file 13 — RAP application directory. Sets up directory for RAP (RDF application for PHP) source code [file 1472-6947-7-3-S13.gz › rap/doc/phpdoc/__filesource/fsource_model__modelModel.php.html]

xml version="1.0" encoding="iso-8859-1"?


File Source for Model.php


# Source for file Model.php

Documentation is available at Model.php

```
1. <?php

3. // ----------------------------------------------------------------------------------
4. // Class: Model
5. // ----------------------------------------------------------------------------------


9. /**
10. * Abstract superclass of MemModel and DbModel. A model is a programming interface to an RDF graph.
11. * An RDF graph is a directed labeled graph, as described in http://www.w3.org/TR/rdf-mt/.
12. * It can be defined as a set of <S, P, O> triples, where P is a uriref, S is either
13. * a uriref or a blank node, and O is either a uriref, a blank node, or a literal.
14. *
15. * <BR><BR>History:<UL>
16. * <LI>03-29-2005                 : Function visualizeGraph() added (anton1@koestlbacher.de)</LI>
17. * <LI>12-06-2004                : Functions findForward() and getMemModelByRDQL() added. (anton1@koestlbacher.de) </LI>
18. * <LI>10-30-2004                : GRDDL-Support (see http://www.w3.org/TR/grddl/) added. (tobias.gauss@web.de) </LI>
19. * <LI>09-22-2004                 : Function getUniqueResourceURI(): default bNode prefix is used,
20. *                                   if no other is defined. Returns unique URIs, without checking the model first.
21. * <LI>09-07-2004                : Function load improved. (auer@informatik.uni-leipzig.de)</LI>
22. * <LI>03-26-2004                 : _addStatementFromAnotherModel() added
23. *                                 : saveAs() moved to the child classes</LI>
24. * <LI>11-13-2003                 : Function saveAs added
25. * <LI>07-27-2003                : This is an abstract parent class for MemModel and DbModel.
26. *                                 The previous class Model has been renamed to MemModel
27. *
28. *
29. *
30. * @version  V0.9.1
31. * @author Radoslaw Oldakowski <radol@gmx.de>
32. * @author Daniel Westphal <mail@d-westphal.de>
33. *
34. * @package model
35. * @access    public
36. */

38. Class Model extends Object {


41. /**
42. * Base URI of the Model.
43. * Affects creating of new resources and serialization syntax.
44. *
45. * @var     string
46. * @access    private
47. */
48. var $baseURI;

50. /**
51. * Number of the last assigned bNode.
52. *
53. *
54. * @var     integer
55. * @access    private
56. */
57. var $bNodeCount;


61. /**
62. * Notice for people who are used to work with older versions of RAP.
63. *
64. * @throws  PHPError
65. * @access    public
66. */
67. function Model() {

69. $errmsg  = 'Since RAP 0.6 the class for manipulating memory models has been renamed to MemModel.';
70. $errmsg .= '<br>Sorry for this inconvenience.<br>';

72. trigger_error($errmsg, E_USER_ERROR);
73. }


76. /**
77. * Return current baseURI.
78. *
79. * @return  string
80. * @access    public
81. */
82. function getBaseURI()  {

84. return $this->baseURI;
85. }


88. /**
89. * Load a model from a file containing RDF, N3, N-Triples or a xhtml document containing RDF.
90. * This function recognizes the suffix of the filename (.n3 or .rdf) and
91. * calls a suitable parser, if no $type is given as string ("rdf" "n3" "nt");
92. * If the model is not empty, the contents of the file is added to this DbModel.
93. *
94. * @param     string     $filename
95. * @param     string     $type
96. * @param   boolean $stream
97. * @access    public
98. */
99. function load($filename, $type = NULL, $stream=false) {

101. if ((isset($type)) && ($type =='n3') OR ($type =='nt')) {
102. // Import Package Syntax
103. include_once(RDFAPI_INCLUDE_DIR.PACKAGE_SYNTAX_N3);
104. $parser = new N3Parser();
105. }elseif ((isset($type)) && ($type =='rdf')) {
106. // Import Package Syntax
107. include_once(RDFAPI_INCLUDE_DIR.PACKAGE_SYNTAX_RDF);
108. $parser = new RdfParser();
109. }elseif ((isset($type)) && ($type =='grddl')) {
110. // Import Package Syntax
111. include_once(RDFAPI_INCLUDE_DIR.PACKAGE_SYNTAX_GRDDL);
112. $parser = new GRDDLParser();
113. }else {
114. // create a parser according to the suffix of the filename
115. // if there is no suffix assume the file to be XML/RDF
116. preg_match("/\.([a-zA-Z0-9_]+)$/", $filename, $suffix);
117. if (isset($suffix[1]) && (strtolower($suffix[1]) == 'n3' OR strtolower($suffix[1]) == 'nt')){
118. // Import Package Syntax
119. include_once(RDFAPI_INCLUDE_DIR.PACKAGE_SYNTAX_N3);
120. $parser = new N3Parser();
121. }elseif (isset($suffix[1]) && (strtolower($suffix[1]) == 'htm' OR strtolower($suffix[1]) == 'html' OR strtolower($suffix[1]) == 'xhtml')){
122. include_once(RDFAPI_INCLUDE_DIR.PACKAGE_SYNTAX_GRDDL);
123. $parser = new GRDDLParser();
124. }else{
125. // Import Package Syntax
126. include_once(RDFAPI_INCLUDE_DIR.PACKAGE_SYNTAX_RDF);
127. $parser = new RdfParser();}
128. };

130. if(($stream && $type=='rdf')||($stream && $type=='n3'))
131. $temp=&$parser->generateModel($filename,false,$this);
132. else{
133. $temp=&$parser->generateModel($filename);
134. }
135. $this->addModel($temp);
136. if($this->getBaseURI()== null)
137. $this->setBaseURI($temp->getBaseURI());

139. }


142. /**
143. * Adds a statement from another model to this model.
144. * If the statement to be added contains a blankNode with an identifier
145. * already existing in this model, a new blankNode is generated.
146. *
147. * @param     Object Statement   $statement
148. * @access    private
149. */
150. function _addStatementFromAnotherModel($statement, &$blankNodes_tmp) {

152. $subject = $statement->getSubject();
153. $object = $statement->getObject();

155. if (is_a($subject, "BlankNode")) {
156. $label = $subject->getLabel();
157. if (!array_key_exists($label, $blankNodes_tmp))
158. {
159. if ($this->findFirstMatchingStatement($subject, NULL, NULL)
160. || $this->findFirstMatchingStatement(NULL, NULL, $subject))
161. {
162. $blankNodes_tmp[$label] = new BlankNode($this);
163. $statement->subj = $blankNodes_tmp[$label];
164. } else {
165. $blankNodes_tmp[$label] = $subject;
166. }
167. } else
168. $statement->subj = $blankNodes_tmp[$label];
169. }

171. if (is_a($object, "BlankNode")) {
172. $label = $object->getLabel();
173. if (!array_key_exists($label, $blankNodes_tmp))
174. {
175. if ($this->findFirstMatchingStatement($object, NULL, NULL)
176. || $this->findFirstMatchingStatement(NULL, NULL, $object))
177. {
178. $blankNodes_tmp[$label] = new BlankNode($this);
179. $statement->obj = $blankNodes_tmp[$label];
180. } else {
181. $blankNodes_tmp[$label] = $object;
182. }
183. } else
184. $statement->obj = $blankNodes_tmp[$label];
185. }
186. $this->add($statement);
187. }


191. /**
192. * Internal method, that returns a resource URI that is unique for the Model.
193. * URIs are generated using the base_uri of the DbModel, the prefix and a unique number.
194. * If no prefix is defined, the bNode prefix, defined in constants.php, is used.
195. *
196. * @param    string    $prefix
197. * @return    string
198. * @access    private
199. */
200. function getUniqueResourceURI($prefix = false)
201. {
202. static $bNodeCount;
203. if(!$bNodeCount)
204. $bNodeCount = 0;

206. if(!$prefix)
207. $prefix=BNODE_PREFIX;

209. return $prefix.++$bNodeCount;
210. }

212. /**
213. * Returns a ResModel with this model as baseModel.
214. *
215. * @param   constant  $modelType
216. * @param   string  $baseURI
217. * @return    object    MemModel
218. * @access    public
219. */
220. function & getResmodel()
221. {
222. require_once( RDFAPI_INCLUDE_DIR . PACKAGE_RESMODEL);
223. return new ResModel($this);
224. }

226. /**
227. * Returns an OntModel with this model as baseModel.
228. * $vocabulary has to be one of the following constants (currently only one is supported):
229. * RDFS_VOCABULARY to select a RDFS Vocabulary.
230. * You can supply a base URI
231. *
232. * @param   constant  $vocabulary
233. * @return    object    OntModel
234. * @access    public
235. */
236. function & getOntModel($vocabulary)
237. {
238. require_once( RDFAPI_INCLUDE_DIR . PACKAGE_ONTMODEL);
239. return new OntModel(& $this, $vocab);
240. }


244. /**
245. * Searches for triples using find() and tracks forward blank nodes
246. * until the final objects in the retrieved subgraphs are all named resources.
247. * The method calls itself recursivly until the result is complete.
248. * NULL input for subject, predicate or object will match anything.
249. * Inputparameters are ignored for recursivly found statements.
250. * Returns a new MemModel or adds (without checking for duplicates)
251. * the found statements to a given MemModel.
252. * Returns an empty MemModel, if nothing is found.
253. * !!!!!!!!!!!!!!!!!!!!!!!!!!!!!!!!!!!!!!!!!!!!!!!!!!!!!!!!!!!!!!!!!!!!!!
254. * WARNING: This method can be slow with large models.
255. * NOTE:    Blank nodes are not renamed, they keep the same nodeIDs
256. *          as in the queried model!
257. * !!!!!!!!!!!!!!!!!!!!!!!!!!!!!!!!!!!!!!!!!!!!!!!!!!!!!!!!!!!!!!!!!!!!!!
258. *
259. * @author   Anton K�stlbacher <anton1@koestlbacher.de>
260. * @param    object Node     $subject
261. * @param    object Node     $predicate
262. * @param    object Node     $object
263. * @param    object MemModel $object
264. * @return   object MemModel
265. * @access   public
266. * @throws   PhpError
267. */

269. function findForward($subject, $predicate, $object, $newModel = NULL)
270. {
271. if (!is_a($newModel, "MemModel"))
272. {
273. $newModel = New MemModel;
274. }

276. if (is_a($this, "DbModel"))
277. {
278. $model = $this;
279. $res   = $model->find($subject, $predicate, $object);
280. $it    = $res->getStatementIterator();
281. }
282. elseif (is_a($this, "MemModel")) {
283. $model = $this;
284. $it    = $model->findAsIterator($subject, $predicate, $object);
285. }
286. elseif (is_a($this, "ResModel")) {
287. $model = $this->model;
288. $it    = $model->findAsIterator($subject, $predicate, $object);
289. }

291. while ($it->hasNext())
292. {
293. $statement = $it->next();
294. $newModel->add($statement);
295. if (is_a($statement->object(),'BlankNode'))
296. {
297. $model->findForward($statement->object(), NULL, NULL,&$newModel);
298. }
299. }
300. return $newModel;
301. }


304. /**
305. * Perform an RDQL query on this Model. Should work with all types of models.
306. * This method returns a MemModel containing the result statements.
307. * If $closure is set to TRUE, the result will additionally contain
308. * statements found by the findForward-method for blank nodes.
309. * !!!!!!!!!!!!!!!!!!!!!!!!!!!!!!!!!!!!!!!!!!!!!!!!!!!!!!!!!!!!!!!!!!!!!!
310. * WARNING: If called with $closure = TRUE this method
311. *          can be slow with large models.
312. * !!!!!!!!!!!!!!!!!!!!!!!!!!!!!!!!!!!!!!!!!!!!!!!!!!!!!!!!!!!!!!!!!!!!!!
313. *
314. * @author   Anton K�stlbacher <anton1@koestlbacher.de>
315. * @author   code snippets taken from the RAP Netapi by Phil Dawes and Chris Bizer
316. * @access   public
317. * @param    string $queryString
318. * @param    boolean $closure
319. * @return   object MemModel
320. *
321. */

323. function & getMemModelByRDQL($queryString, $closure = FALSE)
324. {
325. require_once(RDFAPI_INCLUDE_DIR.PACKAGE_RDQL);
326. $parser = new RdqlParser();
327. $parsedQuery =& $parser->parseQuery($queryString);

329. // If there are variables used in the pattern but not
330. // in the select clause, add them to the select clause
331. foreach ($parsedQuery['patterns'] as $n => $pattern)
332. {
333. foreach ($pattern as $key => $val_1)
334. {
335. if ($val_1['value']{0}=='?')
336. {
337. if (!in_array($val_1['value'],$parsedQuery['selectVars']))
338. {
339. array_push($parsedQuery['selectVars'],$val_1['value']);
340. }
341. }
342. }
343. }

345. if (is_a($this, "DbModel"))
346. {
347. $engine = new RdqlDbEngine();
348. $model  = $this;
349. }
350. elseif (is_a($this, "MemModel"))
351. {
352. $engine = new RdqlMemEngine();
353. $model  = $this;
354. }
355. elseif (is_a($this, "ResModel"))
356. {
357. $engine = new RdqlMemEngine();
358. $model  = $this->model;
359. }

361. $res = $engine->queryModel($model,$parsedQuery,TRUE);
362. $rdqlIter = new RdqlResultIterator($res);
363. $newModel = new MemModel();

365. // Build statements from RdqlResultIterator
366. while ($rdqlIter->hasNext()) {
367. $result = $rdqlIter->next();
368. foreach ($parsedQuery['patterns'] as $n => $pattern)
369. {
370. if (substr($pattern['subject']['value'], 0, 1) == '?')
371. {
372. $subj = $result[$pattern['subject']['value']];
373. }
374. else
375. {
376. $subj = new Resource($pattern['subject']['value']);
377. }
378. if (substr($pattern['predicate']['value'], 0, 1) == '?')
379. {
380. $pred = $result[$pattern['predicate']['value']];
381. }
382. else
383. {
384. $pred = new Resource($pattern['predicate']['value']);
385. }

387. if (substr($pattern['object']['value'], 0, 1) == '?')
388. {
389. $obj = $result[$pattern['object']['value']];
390. }
391. else
392. {
393. if (isset($pattern['object']['is_literal']))
394. {
395. $obj = new Literal($pattern['object']['value']);
396. $obj->setDatatype($pattern['object']['l_dtype']);
397. $obj->setLanguage($pattern['object']['l_lang']);
398. }
399. else
400. {
401. $obj = new Resource($pattern['object']['value']);
402. }
403. }

405. $statement = new Statement($subj,$pred,$obj);
406. $newModel->add($statement);

408. // findForward() Statements containing an eventually given blank node
409. // and add them to the result, if closure = true
410. if (is_a($statement->object(),'BlankNode') && $closure == True)
411. {
412. $newModel = $model->findForward($statement->object(),NULL,NULL,&$newModel);
413. }
414. if (is_a($statement->subject(),'BlankNode') && $closure == True)
415. {
416. $newModel = $model->findForward($statement->subject(),NULL,NULL,&$newModel);
417. }
418. }
419. }
420. return $newModel;
421. }

423. /**
424. * Alias for RDFUtil::visualiseGraph(&$model, $format, $short_prefix)
425. *
426. * !!!!!!!!!!!!!!!!!!!!!!!!!!!!!!!!!!!!!!!!!!!!!!!!!!!!!!!!!!!!!!!!!!!!!!
427. * Note: See RDFUtil for further Information.
428. * !!!!!!!!!!!!!!!!!!!!!!!!!!!!!!!!!!!!!!!!!!!!!!!!!!!!!!!!!!!!!!!!!!!!!!
429. *
430. * @author   Anton K�stlbacher <anton1@koestlbacher.de>
431. * @param    string  $format
432. * @param    boolean $short_prefix
433. * @return   string, binary
434. * @access   public
435. * @throws   PhpError
436. */

438. function visualize($format = "dot", $short_prefix = TRUE)
439. {
440. return RDFUtil::visualizeGraph($this, $format, $short_prefix);
441. }
442. } // end: Model

444. ?>
```

Documentation generated on Thu, 7 Jul 2005 13:42:04 +0200 by phpDocumentor 1.3.0RC3
